# A Case of Metastatic Renal Cell Cancer Presenting as Jaundice

**DOI:** 10.4021/wjon247w

**Published:** 2010-11-02

**Authors:** MinYuen Teo, Barbara Ryan, Niall Swan, Ray S. McDermott

**Affiliations:** a Department of Medical Oncology, AMNCH, Tallaght, Dublin, Ireland; b Department of Gastroenterology, AMNCH, Tallaght, Dublin, Ireland; c Department of Pathology, AMNCH, Tallaght, Dublin, Ireland; d Department of Medical Oncology, Mercy University Hospital, Cork, Ireland

**Keywords:** Kidney cancer, Metastasis, Complication

## Abstract

Renal cell carcinoma is the second most common urological malignancy and it runs a highly variable clinical course. We describe a case of metastatic renal cell carcinoma in a 50-year-old lady with metastasis to the ampulla of Vater, clinically masquerading as cholelithiasis and biliary colic. The clinical, radiographic and endoscopic findings are presented. Ampullary metastases are rare, and prompt recognition and intervention are necessary before patient's performance status is compromised.

## Introduction

Renal cell cancer is the second most common urological malignancy, accounting for 3% of cancers diagnosed worldwide [1]. According to the SEER database, up to 37% of patients have distant disease at diagnosis, while it is estimated that 20-40% of localized tumors will eventually metastasize [2]. Metastatic renal cell carcinoma is notorious for its variable clinical course. Common sites of metastatic disease include lungs, bones, lymph nodes and liver [3, 4]. The proposed routes of dissemination are not dissimilar from a wide range of other solid tumors, namely hematogenous, lymphatic, transcoelomic or by direct invasion. We herein report a case of metastatic renal cell carcinoma metastasizing to the ampulla of Vater, clinically mimicking cholelithiasis and biliary colic.

## Case Report

A 50-year-old lady with a background of obesity, angina and hypercholesterolemia developed a left sided renal cell carcinoma in September 2007. She underwent embolisation followed by radical nephrectomy. Pathological staging showed a pT2, Fuhrman Grade 3 tumor of clear cell type. There was no evidence of metastatic disease at that time and she went on surveillance. In July 2008, she developed pulmonary metastases, and in view of her cardiac history she was commenced on Sorafenib. After 6 months, her restaging computed tomography (CT) scan in Jan 2009 revealed significant interval enlargement of her pulmonary metastases. Her treatment was switched to Sunitinib. Repeat imaging in May 2009 again displayed on-going disease progression in the lungs with no other extrapulmonary visceral involvement. She was therefore commenced on the oral mTOR inhibitor, Everolimus as third line treatment.

**Figure 1:**
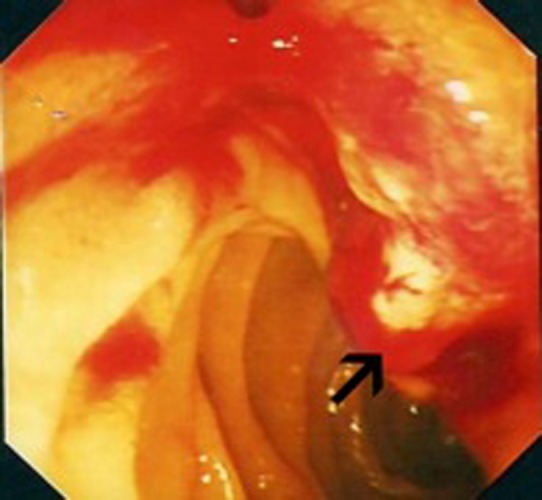
Endoscopic view: black arrow pointing to bleeding mass at the ampulla of Vater.

In July 2009, she presented to the Oncology Day Unit with one week history of obstructive jaundice and a two-day history of right upper quadrant colicky pain. Liver function tests were grossly abnormal with a cholestatic picture, (Alk Phos 750 IU/L, ALT 598 IU/L, γGT 1246 IU/L, bilirubin 228 µmol/L). Initial ultrasonographic assessment proved difficult because of her body habitus, but did suggest ductal dilatation with two areas of low density in the left lobe of the liver. Further imaging in the form of magnetic resonance cholangiopancreaticogram (MRCP) showed high grade intrahepatic bile duct dilatation with a dilated common bile duct to its mid portion, while the pancreatic duct was of normal calibre. There was a suggestion of stricture at the distal end of the common bile duct. Endoscopic examination revealed a swollen ampulla (Fig. 1), and histopathological examination showed fragments of ulcerated small intestinal mucosa extensively infiltrated by poorly differentiated carcinoma with focal clear cell morphology noted. The tumor cells stained positive for pancytokeratin AE1/AE3, vimentin, EMA and CD10 while negative for CK7, CK20 and CDX2. RCC was non-contributory due to excess background staining. The above immunohistochemical findings are most consistent with a metastasis from this lady’s known renal cell carcinoma.

**Figure 2:**
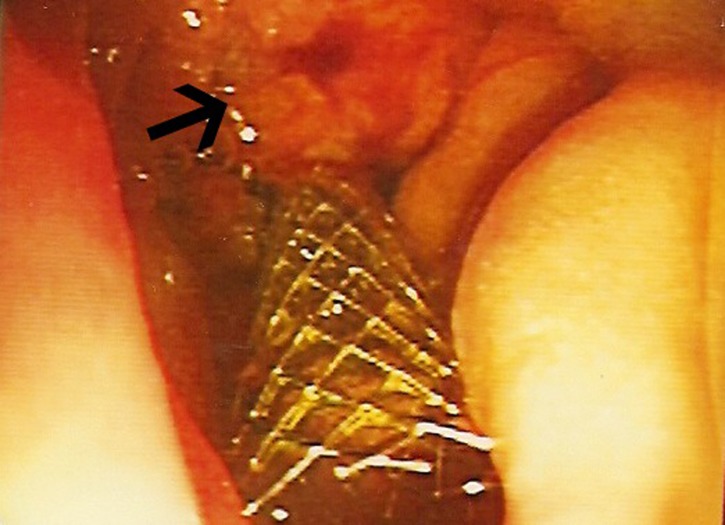
Endoscopic view: black arrow pointing to mass at ampulla of Vater, metallic stent in-situ.

**Figure 3:**
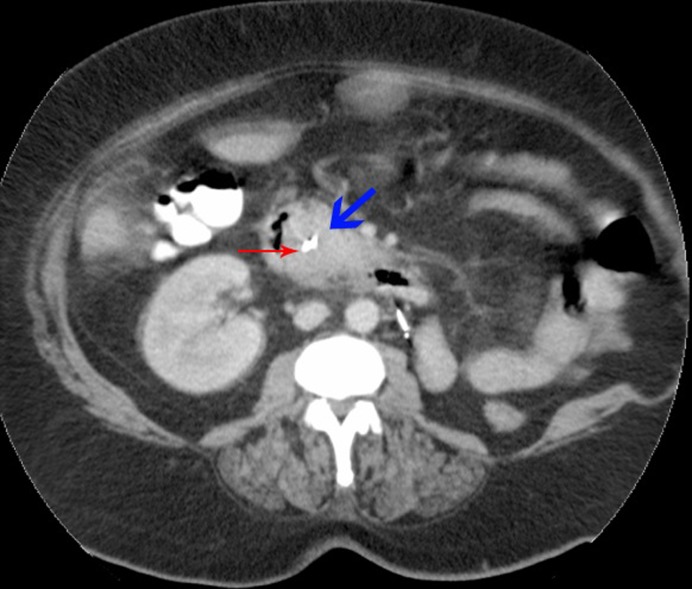
Cross-section of CT abdomen: blue arrow - tumor deposit; red arrow - biliary stent, D2 of duodenum to the right.

**Figure 4:**
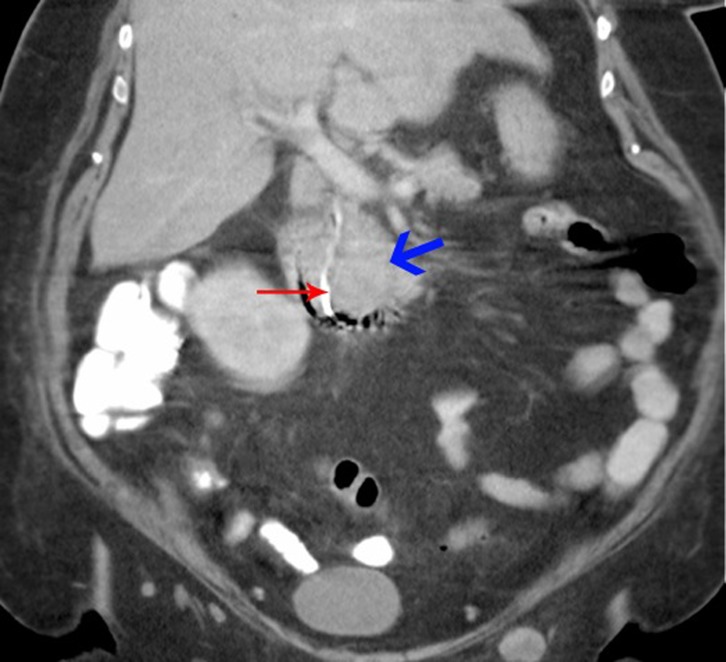
Coronal view of CT abdomen: Blue arrow - tumor deposit; red arrow - biliary stent leading into duodenum.

A biliary stent was inserted successfully during the endoscopic retrograde cholangiopancreaticogram (ERCP) (Fig. 2) and satisfactory drainage was achieved. Her liver enzymes and bilirubin improved significantly. A restaging CT showed a 6.0 x 4.5 cm mass involving the uncinate process of the pancreas and second part of the duodenum (Fig. 3, 4). New liver lesions were also seen.

Shortly thereafter, the patient developed melena, requiring multiple transfusion of red cells. Endoscopic examination showed that the ampullary tumor was infiltrative and bleeding. This was photocoagulated and hemostasis was achieved. In view of the advanced stage of her disease and her deteriorating performance status, she was referred on to the palliative care service and discharged home in September 2009. She died a week later.

## Discussion

This is a case of metastatic renal cell carcinoma to the ampulla, in which extra-pulmonary disease first mimicked cholelithiasis and jaundice. There have previously been reports of renal cell cancer metastasizing to the small intestines, usually mediated by direct tumor invasion of small intestines by the right kidney due to their proximity. On the other hand, duodenal and periampullary metastases appear to be a less common intestinal manifestations [4, 5]. There have been a handful of case reports delineating ampullary metastasis in the context of renal cell cancer [6-8] and a marginally greater number of literatures reporting on duodenal metastasis. These reported cases of ampullary disease uniformly manifested as gastrointestinal bleeding [4, 9], while one case presented as malasorption [10]. The majority of duodenal and ampullary metastases reported have been treated surgically, either with a Whipple’s procedure or variants of it, with varying degrees of success [8]. The above-described patient had multiple co-morbidities and a poor performance status, as well as heavy disease burden, precluding surgical intervention. However, palliative measures such as biliary stenting as adopted in this case could offer symptomatic relief from hepatic congestion. Other symptoms such as gastrointestinal bleeding or obstruction could be managed with endoscopic photocoagulation or stenting, respectively.

The natural history of metastatic renal cell carcinoma can be complicated by uncommon site of metastatic deposit. Ampullary metastases are rare but carry significant morbidity which translates into poorer performance status and hence worse outcome. 
